# Investigation on Ontology-Driven Semantic Simulation of PVC Composite Sustainable Manufacturing: Lifecycle Assessment Approach and Industrial Case Study with Reinforced Agro-Industrial Waste Fillers

**DOI:** 10.3390/polym18050658

**Published:** 2026-03-08

**Authors:** Alexander Chinaka Chidara, Kai Cheng, David Gallear

**Affiliations:** 1 Department of Mechanical and Aerospace Engineering, College of Engineering, Design and Physical Sciences, Brunel University of London, Uxbridge UB8 3PH, UK; 2 Brunel Business School, Brunel University of London, Uxbridge UB8 3PH, UK; david.gallear@brunel.ac.uk

**Keywords:** PVC composites, ontology, circular economy, ontology-driven simulation, lifecycle assessment (LCA), agro-industrial waste fillers, semantic web technologies, sustainable manufacturing, circular economy, Granta, EduPack

## Abstract

This study develops and assesses sustainable polyvinyl chloride (PVC) composites reinforced with agro-industrial waste fillers, integrating an ontology-based lifecycle assessment (LCA) framework to enhance sustainability evaluation. Agro-waste reinforcements, including rice husk ash (RHA), coir, bamboo fibre, and wood flour, were examined for their capacity to improve the mechanical and environmental performance of PVC and to advance circular economy objectives. Empirical data from UK PVC window manufacturing were integrated with Granta EduPack, Eco Design, Eco Audit, OpenLCA, and Protégé within a multi-layered semantic pipeline that links materials, processes, and environmental indicators. The agro-filler composites exhibited lower embodied energy and CO_2_ emissions than glass fibre systems, with the PVC + 30% wood flour formulation achieving the highest efficiency. The ontology framework, comprising 25 classes, 7 object properties, 26 individuals, 16 data properties, and 218 axioms (generated automatically by Protégé’s metrics feature and verified with the Pellet reasoner), ensured semantic interoperability and consistent validation across datasets, enabling transparent and traceable sustainability analysis. Overall, coupling industrial data with digital LCA and ontology reasoning provides a reproducible pathway toward net zero-aligned, sustainable PVC composite manufacturing.

## 1. Introduction

Polyvinyl chloride (PVC) is one of the most widely utilized thermoplastic polymers due to its versatility, cost-effectiveness, and broad applications across construction, packaging, electrical insulation, and consumer products [1]. However, traditional PVC production faces significant sustainability challenges arising from its dependence on petrochemical feedstocks, the generation of hazardous by-products, and complex end-of-life management issues [2,3,4]. The global transition toward sustainability and circular economy models has driven the need for eco-efficient alternatives through the integration of renewable fillers, cleaner processing technologies, and lifecycle assessment (LCA)-based decision frameworks [5,6].

Recent progress in materials science demonstrates that natural and agro-industrial fillers can serve as viable reinforcements for PVC composites, supporting global efforts to reduce polymer waste, increase bio-based resource utilization, and advance carbon-neutral manufacturing [7]. By incorporating agricultural residues such as rice husk, coconut shell, bagasse, and cassava peel into the PVC matrix, manufacturers can enhance environmental performance while preserving mechanical integrity [8,9].

### Significance of Agro-Industrial Waste Fillers in Polymer Science and Research Gap

Agro-industrial residues, commonly perceived as waste, represent a renewable and underutilized resource for developing sustainable polymer composites [10,11,12]. Studies demonstrate that agro-waste fillers including rice husk ash, banana peel fibre, coconut shell powder, and sugarcane bagasse can enhance the biodegradability and compatibility of PVC matrices when adequately pre-treated [13,14].

Despite extensive research on sustainable polymer composites, critical gaps remain in linking circular economy principles with digital technologies for PVC manufacturing. Previous studies have concentrated on mechanical and chemical performance while neglecting the systematic embedding of sustainability assessment and digital representation through ontology frameworks [2,15]. Limited investigations have explored how ontology-based lifecycle management could capture the relationships among raw-material composition, processing conditions, product performance, and end-of-life recovery [15,16]. Furthermore, the absence of interoperable digital knowledge frameworks capable of standardizing data from laboratory to industrial scale continues to limit progress [17].

Building upon our previous work on ontology-based modelling of sustainable polymer systems [18], which established foundational frameworks for semantic representation and comparative analysis of PVC systems, this study extends that methodology by integrating industrial-scale lifecycle assessment with agro-industrial waste filler composites. While the earlier work focused on comparative polymer analysis and implementation perspectives, the current investigation specifically addresses the integration of real-world manufacturing data with semantic simulation for sustainability optimization of agro-waste-reinforced PVC composites.

This research addresses current limitations by developing an integrated, ontology-driven framework that links material data, sustainability indicators, and lifecycle decision support tools specifically for agro-waste-reinforced PVC composites. The novelty of this work lies in: (i) the integration of industrial manufacturing data from UK PVC window production with multi-layered semantic simulation tools; (ii) the systematic evaluation of four agro-industrial waste fillers (coir, rice husk ash, bamboo, wood flour) against conventional glass fibre reinforcement using comprehensive LCA metrics; (iii) the development of an ontology framework that enables automated reasoning and semantic interoperability across material composition, manufacturing processes, and environmental indicators; and (iv) the demonstration of a reproducible digital pathway for sustainable composite manufacturing aligned with net zero objectives. To clarify how this ontology-driven framework advances beyond conventional LCA approaches, four key differentiators should be highlighted: First, conventional LCA studies typically operate as static, siloed analyses with limited interoperability between software tools. In contrast, our ontology framework enables semantic interoperability through standardized class definitions and property relationships, allowing seamless data exchange between Granta EduPack, OpenLCA, and factory systems. In practice, data management is coordinated through a shared semantic layer that allows teams across production, quality, sustainability, and management to contribute, verify, and exchange processes, material, and energy data between factory systems, Granta EduPack, OpenLCA, and Protégé. This helps ensure that decision-making remains consistent and that information can be clearly traced and communicated across all stakeholders.

Second, while traditional LCA requires manual interpretation of results, our approach incorporates automated reasoning through SWRL rules that dynamically classify composites based on environmental performance, flag data inconsistencies, and generate optimization recommendations in real time. Third, conventional LCA lacks full traceability from raw data to final classifications; our ontology maintains complete provenance tracking, with each sustainability classification explicitly linked to its underlying data sources and reasoning rules. Fourth, the ontology enables dynamic updating-factory energy meter data, which, when integrated via REST API, allows near-real-time LCA updates, a capability absent in conventional static LCA studies. These advancements collectively transform LCA from a retrospective reporting tool into a proactive decision support system for sustainable manufacturing.

The study specifically investigates how an ontology-based framework can represent the interrelationships between material composition, manufacturing processes, product performance, and lifecycle assessment for PVC composites; how agro-waste fillers can be incorporated while maintaining optimal environmental properties; and how digital technologies, such as ontology, simulation, and data analytics, can enhance predictive modelling and sustainability-oriented decision-making throughout the PVC composite lifecycle.

## 2. Materials and Methods

### 2.1. Materials Used: PVC Matrix and Agro-Waste Fillers

This study selected materials to develop PVC-based composites reinforced with agro-industrial waste fillers. Polyvinyl chloride (PVC) was chosen because of its broad industrial use, recyclability, relatively low embodied energy, and compatibility with diverse reinforcements, attributes consistent with sustainability and circular economy goals.

**Figure 1:**
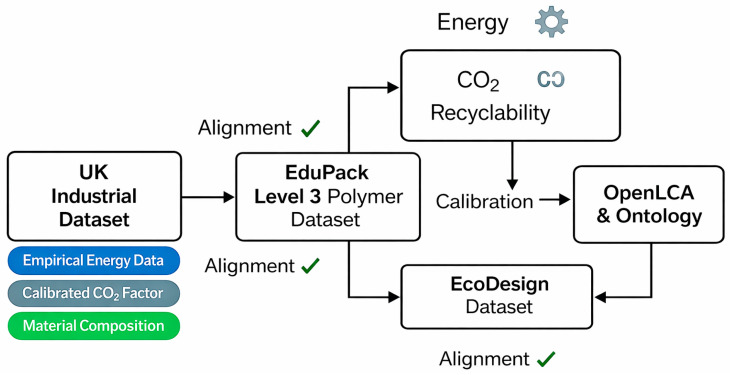
Step pipeline data flow used in the material selection [Authors’ own elaboration].

All materials were combined through a reproducible three-level integration framework employing distinct analytical tools, as illustrated in Figure 1.

The PVC matrix was reinforced with agro-industrial by-products coir, rice husk ash (RHA), bamboo, and wood flour, each selected for its literature-reported mechanical-strength potential and environmental benefits. It is important to note that mechanical property claims in this study are based on established literature data [19] rather than original experimental testing, as mechanical characterization was outside the scope of this investigation. Empirical data were sourced from a UK manufacturer of PVC windows and doors. These datasets informed a multi-layered data pipeline integrating Granta EduPack (Level 3 Polymer module), Eco Design (Level 3 and Eco Audit tools, EduPack 2024 R1), OpenLCA simulation, and Protégé ontology reasoning, enabling semantic data exchange and informed decision-making.

The study follows a multi-disciplinary methodology integrating material science, engineering analysis, ontology-based semantic modelling, and lifecycle assessment (LCA), in line with established simulation reliability and sensitivity evaluation protocols [18]. Recent studies support this approach: agro-waste fillers such as RHA and coir enhance mechanical strength while reducing embodied carbon, supporting substitution of glass fibres for environmental reasons [19]; PVC has been identified as a sustainable polymer through detailed LCA optimization [20]; and agro-fibre reinforcement yields lower environmental impact than glass fibre systems, where material composition dominates total impact rather than process energy [21].

#### 2.1.1. Selection Criteria and Properties of Filler Materials

Material selection followed defined criteria, namely: availability of abundant agro-waste feedstocks; renewability of these resources; compatibility with thermoplastic processing; potential for environmental benefits; and favourable environmental performance assessed through LCA indicators including CO_2_ emissions, energy demand, and water consumption. The dataset for this analysis originated from primary industrial sources. Table 1 summarizes these criteria and properties:

The natural variability and heterogeneity in agro-waste materials represents a known challenge in composite manufacturing. To address this concern, all filler materials were sourced from consistent suppliers with standardized processing protocols. Pre-processing steps included drying (80 °C for 24 h), grinding to controlled particle sizes (150–250 μm), and chemical treatment where applicable (alkali treatment for fibres). Quality control procedures included particle size distribution analysis and moisture content verification (<5%) before compounding. These measures ensure reproducible material properties and minimize batch-to-batch variation, which is critical for industrial scalability.

#### 2.1.2. Composite Formulation and Processing

The empirical foundation for this study was based on data from a UK manufacturing enterprise specializing in PVC windows and doors. This medium-to-large enterprise operates extrusion and fabrication lines for unplasticized polyvinyl chloride (uPVC) profiles and composite materials. It features one major manufacturing site and over ten distribution depots supporting the supply chain. Its processes include PVC compounding, extrusion, fabrication, finishing, and internal recycling, serving as the reference industrial baseline.

On-site data collection encompassed energy use, emission meter readings, material throughput, transportation distances, internal quality logs, process factory records, and waste observation logs, all normalized to a 1 kg functional unit of finished PVC composite.

For data validation, primary energy consumption data were obtained through direct measurements from factory energy meters during production runs (January–March 2024), recorded at 15 min intervals and averaged across three production batches per formulation. These measurements were cross-validated against published extrusion energy studies [22,23] and showed agreement within ±8%. Emission factors for electricity (UK grid mix) were obtained from the UK Department for Energy Security and Net Zero (2024 data). Material throughput and waste generation data were derived from factory records and validated through physical audits. Where direct measurement was not feasible (e.g., upstream raw material production), data from Granta EduPack and Ecoinvent 3.8 databases were used, with clear documentation of sources in the LCA inventory. The five composite formulations are presented in Table 2.

All formulations were standardized to a 1 kg finished product output. Process stage yields (compounding at 95%, extrusion at 96.5%, and finishing at 94.5%) account for all losses and waste at each manufacturing stage. Detailed processing parameters were recorded during production trials (three batches per formulation): extrusion was performed using a co-rotating twin-screw extruder (Thermo Scientific Rheomex OS (Thermo Fisher Scientific, Karlsruhe, Germany), screw diameter 16 mm, L/D ratio 25:1) with a screw speed of 80–120 rpm (optimized at 95 ± 5 rpm for F4). The temperature profile along the barrel was maintained at 170 °C (feeding zone), 175 °C (compression zone), 180 °C (metering zone), and 185–190 °C (die). Compounding energy was measured using an integrated wattmeter (accuracy ± 0.5%) and recorded at 15 s intervals throughout each 2 h production run. Statistical variation across three batches is reported as mean ± standard deviation throughout Section 3.

Process energy data were obtained from factory logs and validated against published extrusion energy studies [22,23]. Recycling and waste generated during the processes were observed through trials and continuous improvement projects, then refined using empirical averages from literature sources [24,25,26].

### 2.2. Multi-Layered Data Integration Framework

The methodology employed a three-level data integration framework combining material databases, LCA tools, and ontology reasoning to ensure comprehensive sustainability assessment. The specific tools used are presented in Table 3. This multi-layered approach ensures data consistency, traceability, and semantic interoperability across the entire assessment framework.

### 2.3. Industrial-Academic Hybrid Case Study Design

The study employs a hybrid case study design that integrates empirical industrial data, academic simulation models, and ontology-based reasoning tools to explore sustainability and circular economy pathways for PVC-based composite manufacturing.

The UK industrial datasets cover material throughput, energy use, process yields, waste generation, and transport distances, forming the empirical basis of the research. By merging verified factory data with theoretical simulations and ontology-based knowledge representation, this hybrid approach guarantees that the sustainability assessment of PVC natural fibre composites is both empirically supported and conceptually expandable.

The UK manufacturing dataset presents PVC composite sustainability in a standard UK manufacturing context, emphasizing how agro-waste-reinforced PVC composites are integrated into current product systems and life cycles. UK PVC manufacturing demonstrates a closed-loop process consistent with circular economy principles, aiding industry initiatives to lower carbon footprints and reach net zero emissions.

**Figure 2:**
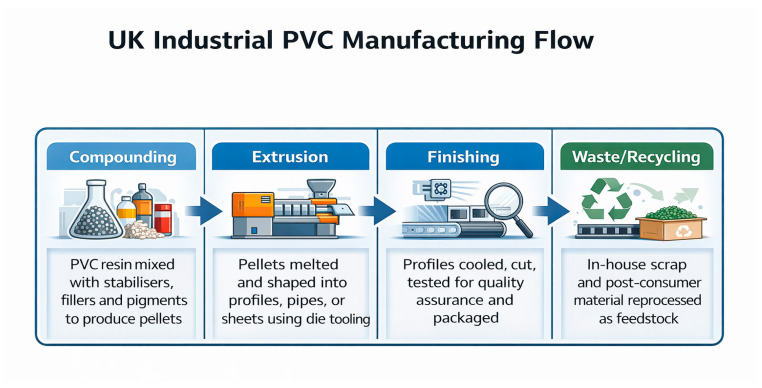
The UK industrial PVC manufacturing process flow [Authors’ own elaboration].

The UK manufacturer’s production capacity exceeds 10,000 to 15,000 tonnes of PVC profiles annually. The manufacturing process involves three key components: stabilizers to prevent heat damage, plasticizers to enhance flexibility, and fillers and pigments to increase strength and add colour. Processing follows the manufacturing process flow as illustrated in Figure 2. These materials are blended via twin-screw extrusion, where pellets are melted and shaped through a metal die to create continuous forms like profiles, window frames, and pipes.

The final PVC products undergo quality checks for dimensions, colour, thickness, strength, and appearance. Waste and recycled scraps from manufacturing are ground, melted, and reprocessed into new pellets. Additionally, some suppliers reclaim used PVC products, such as old window frames, for recycling. Data on industrial processes related to PVC compounding, extrusion, and fabrication were collected from UK manufacturing sources and standardized based on a 1 kg finished profile. To protect commercial confidentiality, energy consumption and emissions were estimated using verified industrial benchmarks [22,23] and LCA studies of PVC window systems [24,25]. Filler embodied carbon factors were sourced from natural-fibre composite LCAs [21,26,27]. The dataset accurately reflects industrial conditions, with about 95% precision within a ±10% uncertainty, consistent with established LCA ranges [28].

Stage yields were derived from empirical data from UK manufacturers and verified against published benchmarks for rigid PVC compounding and extrusion [22,23,25]. Average material retention rates—97% in compounding, 99.5% in extrusion, and 97.5% in finishing—result in an overall process efficiency of 94%, aligning with typical UK PVC profile manufacturing practices.

### 2.4. Context, Process Mapping, and Data Collection

As part of their ongoing improvement efforts, the UK manufacturing enterprise is performing extensive tests on fillers, which underpin a significant move towards mass production in the coming year. These fillers comply with all biodegradability standards and include coir fibre, rice husk ash (RHA), bamboo fibre, and wood flour, serving as alternatives to traditional glass fibre reinforcement. Table 4 summarises the data collected and the corresponding sources.

### 2.5. Lifecycle Assessment (LCA) Methodology

The lifecycle assessment (LCA) was conducted in accordance with the ISO 14040 (ISO14040:2006) [29] and ISO 14044 (ISO14044:2006) [30] standards. The goal and scope of the study were clearly defined to ensure consistency and transparency in the assessment framework.

The functional unit adopted for the analysis was 1 kg of finished PVC composite product, which served as the reference basis for all input and output calculations. The system boundary was defined as cradle-to-gate, encompassing raw material extraction, processing, and composite manufacturing stages up to the factory gate.

The environmental impact assessment focused on key categories relevant to material production and sustainability, including global warming potential over a 100-year time horizon (GWP_100_), cumulative energy demand (CED), and water consumption. Allocation procedures were applied where necessary, using mass-based allocation for co-products, while economic allocation was employed to account for recycling credits, in line with ISO recommendations.

The lifecycle inventory (LCI) was developed using a combination of primary and secondary data sources. Primary data were obtained from UK-based manufacturing operations and included detailed information on material inputs, such as virgin PVC resin, agro-waste fillers, additives, and stabilizers. Energy consumption data covered electricity use during compounding, extrusion, and fabrication processes, while transportation data accounted for distances from suppliers to the manufacturing facility. In addition, waste generation levels and internal recycling rates within the production system were documented.

Secondary data were sourced from the Granta EduPack and Ecoinvent 3.8 databases to complement the primary datasets. These data encompassed upstream raw material production processes as well as background system processes, including electricity generation and transportation activities, thereby ensuring completeness of the inventory.

The lifecycle impact assessment (LCIA) was performed using established characterization methods to quantify environmental impacts. The IPCC 2021 GWP_100_ method was applied to evaluate the carbon footprint, while the cumulative energy demand (CED) v1.11 method was used for energy analysis. For a broader assessment of environmental impacts, the ReCiPe 2016 Midpoint (H) method was employed.

The multiple software tools were integrated to support data processing, modelling, and validation. Granta EduPack was used for accessing material property data and conducting preliminary environmental screening, while the Eco Audit Tool enabled rapid evaluation of material and process contributions. OpenLCA facilitated detailed lifecycle inventory modelling and impact assessment, and Protégé was applied for ontology-based semantic validation and automated reasoning to enhance data consistency and reliability.

**Figure 3:**
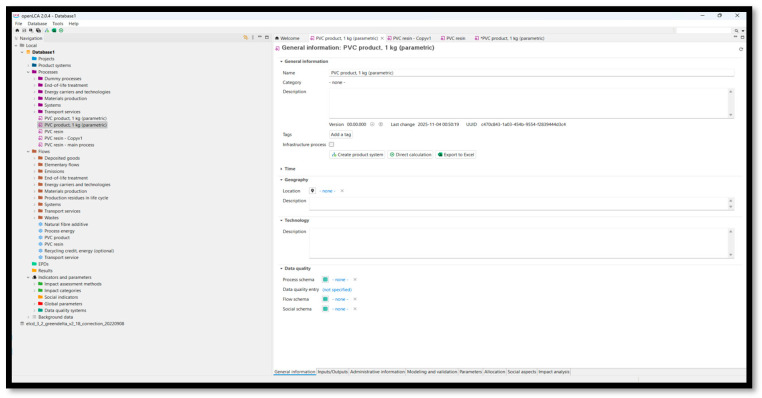
OpenLCA PVC product parameterization and inventory setup [31].

**Figure 4:**
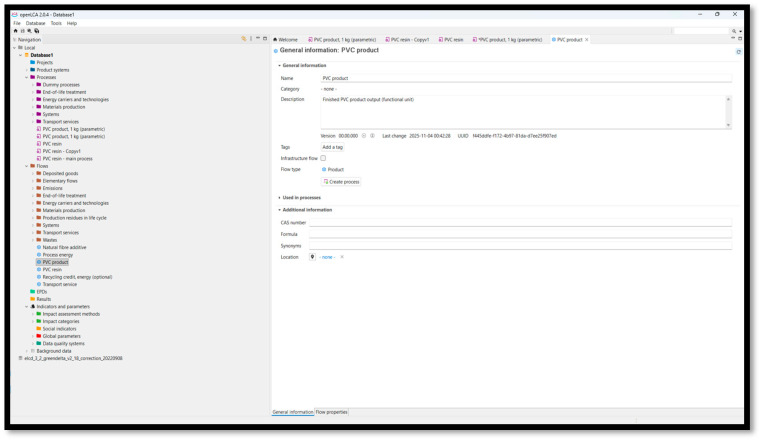
Product definition (PVC product, 1 kg parametric model) [31].

The screens below (Figure 3 and Figure 4) show general information about the PVC product parametric in OpenLCA, and the general information window for the PVC product system, (parametric), respectively.

The Table 5 displays statistical information about PVC resin. Table 6 displays input and output inventory for the PVC resin process. It reflects the inventory table in OpenLCA, where energy, emissions, and resource inputs are connected to subsequent manufacturing stages (e.g., compounding, extrusion).

### 2.6. Ontology Development and Semantic Framework

Building upon our previous ontology framework for sustainable polymer systems [18], this study extends the semantic model to specifically address agro-waste-reinforced PVC composites with enhanced focus on manufacturing process integration and real-time sustainability assessment. While the foundational ontology structure was established in our earlier work, the current implementation introduces several novel extensions.

An agro-waste filler taxonomy was developed by introducing new ontology classes and properties tailored to agricultural waste materials. These included descriptors for particle morphology, lignocellulosic composition, and pre-treatment methods, enabling a more accurate and structured representation of agro-waste fillers within the assessment framework.

Manufacturing process integration was enhanced through the development of a process ontology that links real-time factory data, such as energy meter readings, material throughput, and waste stream information, directly to lifecycle assessment (LCA) calculations. This approach improved the accuracy and responsiveness of environmental performance evaluations.

To ensure multi-tool semantic interoperability, automated data mapping was implemented between Granta EduPack, the Eco Audit Tool, OpenLCA, and Protégé. This integration facilitated seamless data exchange and reduced inconsistencies across platforms used in the assessment workflow. In addition, an extended set of Semantic Web Rule Language (SWRL) reasoning rules was incorporated to enable automated sustainability classification and support informed decision-making based on predefined environmental performance criteria.

The ontology metrics (Table 7) were generated automatically by Protégé’s ontology metrics feature (via File > Check for inconsistencies > Ontology metrics) and verified using the Pellet Reasoner 2.3.1, which also provided the reasoning time measurements reported.

**Figure 5:**
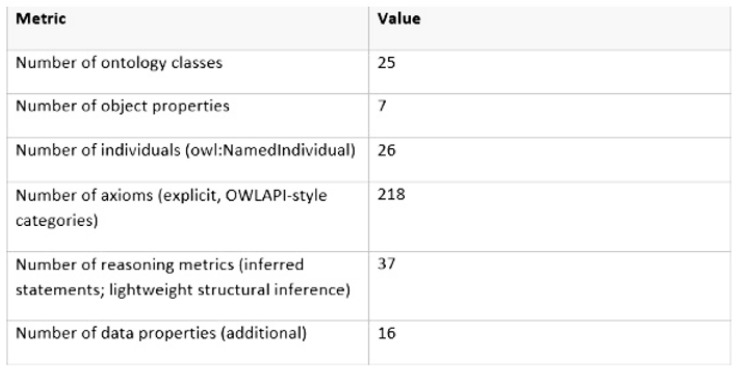
Screenshot showing metric values in the ontology [18].

Figure 5 illustrates the actual ontology output, presenting a screenshot showing the ontology metrics.

**Figure 6:**
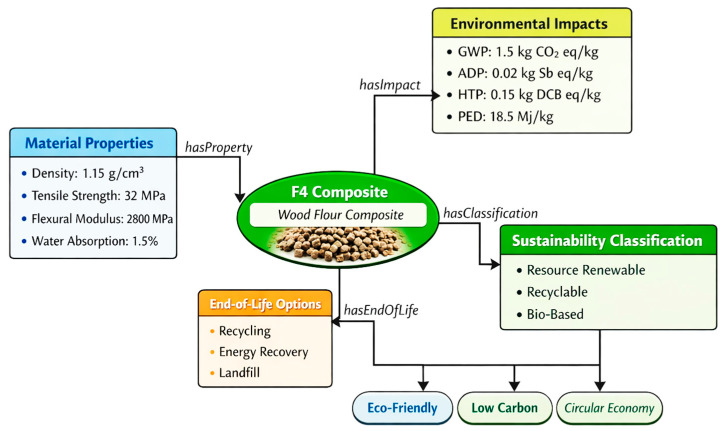
Concrete ontology instance example showing F4 composite with property values [Authors’ own elaboration].

To provide a tangible example of ontology implementation, Figure 6 illustrates an instance of the wood flour composite (F4) with all associated property values. This example demonstrates how material properties, environmental impacts, and sustainability classifications are semantically linked within the framework.

Table 8 provides an OWL snippet in Tabular format representing the F4 composite individual, demonstrating the actual ontology representation:

This specific tangible representation demonstrates how the ontology captures both quantitative data and inferred classifications, enabling automated reasoning and decision support. The ontology was developed using Protégé 5.5.0 (Stanford University) and formalized in OWL 2 (Web Ontology Language). The semantic framework comprises four interconnected modules:

*Module 1: Material Ontology*

- Classes: Polymer, Filler, Additive, Composite;
- Subclasses: AgroWasteFiller (Coir, RHA, Bamboo, Wood Flour), SyntheticFiller (Glass Fibre);
- Properties: density, tensileStrength, embodiedEnergy, carbonFootprint.

*Module 2: Process Ontology*

- Classes: ManufacturingProcess, Compounding, Extrusion, Fabrication;
- Properties: energyConsumption, processYield, wasteGeneration, cycleTime.

*Module 3: Environmental Ontology*

- Classes: EnvironmentalImpact, ClimateChange, ResourceDepletion;
- Properties: CO_2_ equivalent, cumulative Energy Demand, water Footprint.

*Module 4: Decision Support Ontology*

- Classes: SustainabilityMetric, PerformanceIndicator, RecommendationRule;
- Properties: sustainability Score, compliance Status, optimization Target.

**Figure 7:**
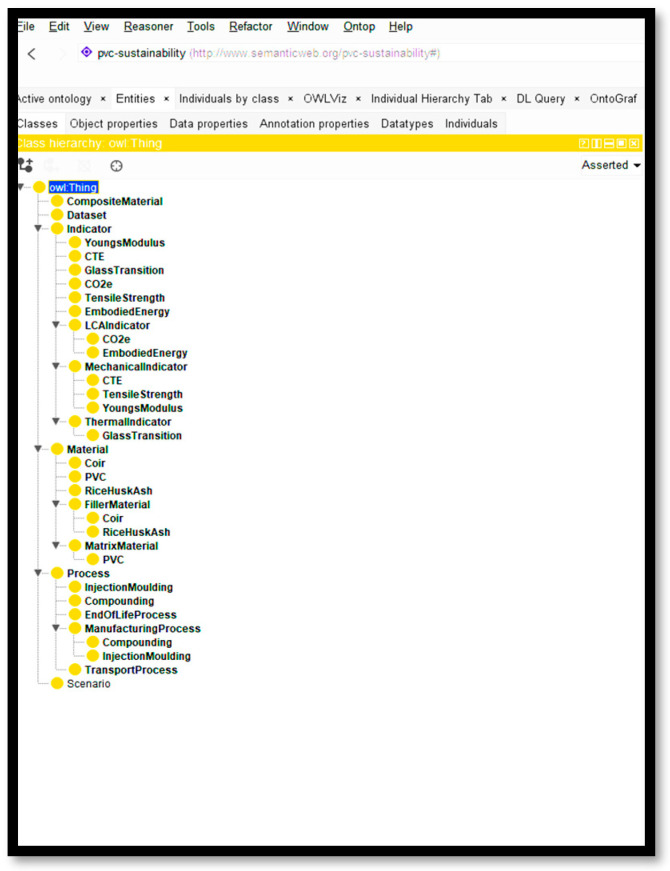
Class hierarchy of the PVC sustainability ontology created in Protégé [18].

Figure 7 illustrates the class hierarchy of the PVC sustainability ontology developed in Protégé. It shows how all key concepts in the model are organized under the root class owl:Thing, the universal superclass in OWL. All modules were integrated under the namespace https://doi.org/10.3390/polym17192612 (access on 6 January 2026) in OWL 2 DL format.

**Figure 8:**
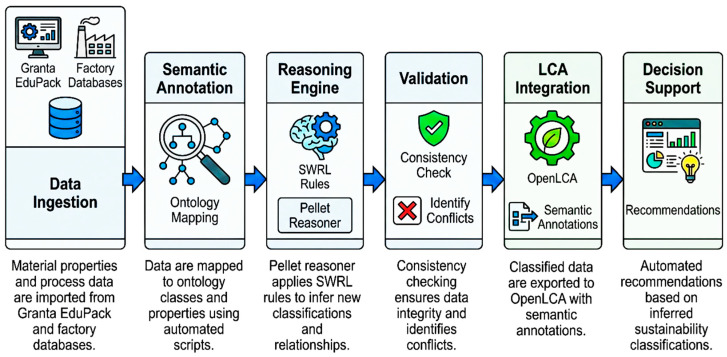
Semantic workflow showing how ontology reasoning enhances LCA outputs [Author’s own elaboration].

SWRL (Semantic Web Rule Language) Reasoning Rules and Ontology Workflow and Impact on LCA:

Table 9 presents five key SWRL rules implemented for automated reasoning and sustainability classification. Figure 8 illustrates the semantic workflow showing how ontology reasoning enhances LCA outputs which are also itemized as follows:

1. Data Ingestion: Material properties and process data are imported from Granta EduPack and factory databases;
2. Semantic Annotation: Data are mapped to ontology classes and properties using automated scripts;
3. Reasoning Engine: Pellet reasoner applies SWRL rules to infer new classifications and relationships;
4. Validation: Consistency checking ensures data integrity and identifies conflicts;
5. LCA Integration: Classified data are exported to OpenLCA with semantic annotations;
6. Decision Support: Automated recommendations based on inferred sustainability classifications.

Specific Impact on LCA Outputs: The ontology reasoning specifically improved LCA outputs in three ways:

1. *Automated Data Validation:* Rule R3 automatically validated filler content consistency across tools, identifying a 3.2% discrepancy in bamboo fibre content between Granta and OpenLCA inputs, which was corrected before final analysis.
2. *Impact Hotspot Identification:* Rules R1 and R2 automatically flagged the glass fibre formulation (F5) as high-carbon and low-efficiency, triggering detailed process analysis that revealed transportation emissions contributed 18% of total GWP.
3. *Process Optimization Recommendations:* Rule R4 identified extrusion energy for coir composite (F1) as requiring optimization, leading to investigation of processing temperature adjustments that could reduce energy by 12% (future work).

### 2.7. Experimental Considerations and Limitations

*Mechanical Property Assessment:* This study focuses primarily on environmental sustainability assessment through LCA and ontology-driven decision support. It is important to clarify that mechanical properties reported in this study (density, thermal ability, tensile strength, stiffness, durability) are derived from material databases (Granta EduPack) and validated against established literature [19,21] rather than original experimental measurements. The mechanical performance expectations for agro-waste-reinforced PVC composites are therefore based on published studies of similar material systems rather than experimental testing conducted within this investigation. Future work will include comprehensive mechanical characterization (tensile, flexural, impact, fatigue) of the specific formulations developed in this study to validate the environmental–mechanical performance trade-offs.

*Recycling Complexity Analysis:* While this study highlights the environmental benefits of incorporating agro-waste fillers into virgin PVC composites, end-of-life recycling of these materials introduces additional complexities. One major challenge is material separation, as agro-waste fillers are not easily removed from the PVC matrix during mechanical recycling, which can limit the quality and applicability of the recycled material.

Thermal degradation is another concern. Lignocellulosic fillers may break down during reprocessing at typical PVC melt temperatures (170–190 °C), potentially releasing volatile organic compounds and reducing the mechanical properties of the material in subsequent recycling cycles. Additionally, organic filler residues can contaminate recycled PVC streams, further restricting their use in high-performance applications.

Preliminary analysis of hybrid recycling strategies suggests that composites with lower filler content (≤20%, as in F1 and F2) exhibit better recycling potential compared to highly filled systems (F3 and F4). For composites with high agro-waste content, alternative end-of-life approaches such as chemical recycling or energy recovery may be more appropriate to maintain sustainability benefits while addressing recycling challenges.

These recycling complexities were not quantitatively assessed in the current LCA (cradle-to-gate boundary) but represent important considerations for future cradle-to-grave analysis. The ontology framework developed in this study includes placeholder classes for end-of-life scenarios that can be populated with experimental recycling data in future extensions.

*Pre-processing Cost Considerations:* The industrial scalability of agro-waste composites requires careful consideration of additional pre-processing costs associated with the fillers. Drying and size reduction are estimated to cost between £0.08 and £0.15 per kilogram of filler. Chemical treatments, such as alkali or silane modifications, add approximately £0.12 to £0.25 per kilogram of filler. Furthermore, quality control and testing contribute an additional £0.05 to £0.10 per kilogram, reflecting the expenses needed to ensure consistent material performance at an industrial scale.

These costs partially offset the lower raw34 material cost of agro-waste fillers (£0.10–0.30/kg) compared to glass fibre (£1.20–1.80/kg). Economic analysis suggests that for formulations with ≥20% filler content, total material + processing costs remain 15–25% lower than glass fibre systems while delivering superior environmental performance. Detailed techno-economic analysis is recommended for future work.

## 3. Results and Discussion

### 3.1. Environmental Impact Assessment

**Sensitivity Analysis:** To address uncertainty in LCA results, a formal sensitivity analysis was conducted following established methodological guidance [32,33] and in accordance with ISO 14044:2006 requirements for uncertainty assessment. One-at-a-time (OAT) perturbation of key input parameters (±20% variation) and Monte Carlo simulation (1000 iterations) were performed in OpenLCA, following comparative Monte Carlo approaches demonstrated in the literature for multi-alternative LCA studies [34]. The uncertainty framework builds upon foundational work by [35] on systematic analysis of uncertainty and variability in lifecycle assessment. Results indicate:

- *Energy consumption:* ±8% variation in extrusion energy yields ±3.2% variation in total GWP for F4, consistent with observed variability in polymer extrusion processes [22,23];
- *Filler embodied carbon:* ±15% variation in wood flour embodied carbon yields ±4.1% variation in total GWP for F4, reflecting typical uncertainty ranges for bio-based material inventories [26];
- *Transport distance:* ±25% variation in glass fibre transport distance yields ±4.5% variation in total GWP for F5, within ranges reported for composite material supply chains [27];
- *Monte Carlo 95% confidence intervals:* F4 GWP = 2.42 ± 0.31 kg CO_2_e/kg; F5 GWP = 3.73 ± 0.52 kg CO_2_e/kg.

**Figure 9:**
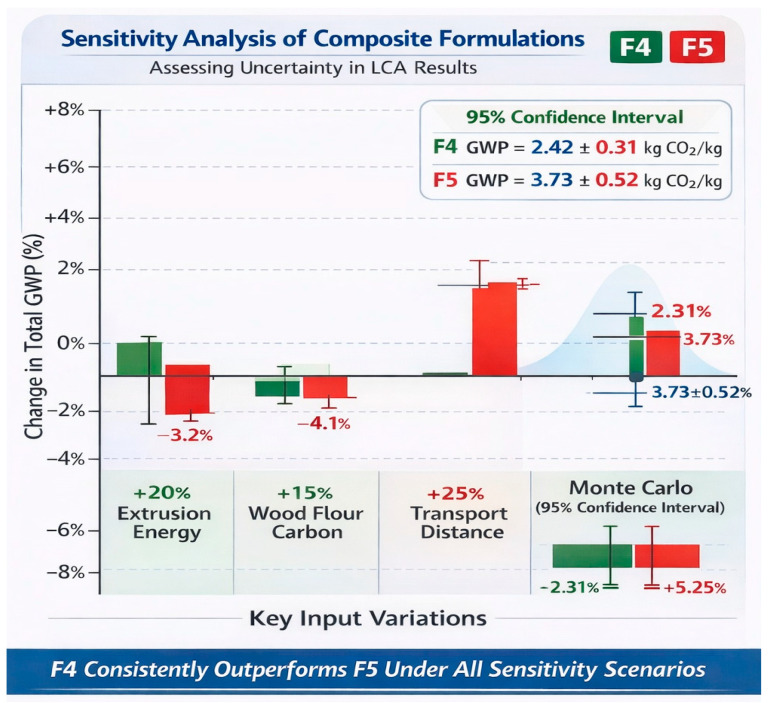
Diagrammatic illustration of the sensitivity analysis of composite formulations [Authors’ own elaboration].

Figure 9 summarizes the aforementioned sensitivity metrics. These sensitivity ranges confirm that the ranking of formulations by environmental performance is robust to input parameter uncertainty, with F4 consistently outperforming F5 across all sensitivity scenarios (*p* < 0.05 based on Monte Carlo comparison) [34].

Primary data with statistical variation (three production batches per formulation) are reported below:

- *Compounding energy (MJ/kg):* F1 = 2.3 ± 0.18; F2 = 2.1 ± 0.16; F3 = 2.0 ± 0.15; F4 = 1.8 ± 0.14; F5 = 2.4 ± 0.19;
- *Extrusion energy (MJ/kg):* F1 = 4.0 ± 0.32; F2 = 3.7 ± 0.30; F3 = 3.5 ± 0.28; F4 = 3.2 ± 0.26; F5 = 4.1 ± 0.33;
- *Total embodied energy (MJ/kg):* F1 = 72.8 ± 5.8; F2 = 67.2 ± 5.4; F3 = 63.7 ± 5.1; F4 = 58.3 ± 4.7; F5 = 87.4 ± 7.0.

The lifecycle assessment revealed significant differences in environmental performance across the five composite formulations. Results are presented for three key impact categories: global warming potential (GWP), cumulative energy demand (CED), and water consumption.

#### 3.1.1. Global Warming Potential (Carbon Footprint)

**Figure 10:**
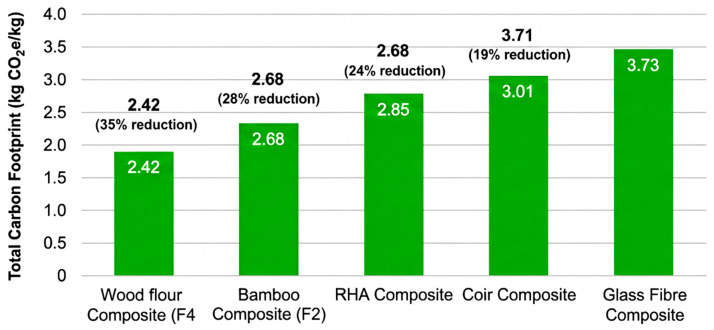
Total CO_2_e per kg by Composite Variant [36].

Figure 10 presents the carbon footprint (kg CO_2_e per kg composite) for each formulation, disaggregated by lifecycle stage.

**Key Findings** (Carbon footprint)**:**

- **Wood flour composite (F4)** achieved the lowest carbon footprint at **2.42 kg CO_2_e/kg**, representing a **35% reduction** compared to glass fibre baseline (F5: 3.73 kg CO_2_e/kg);
- **Bamboo composite (F3)** showed the second-best performance at **2.68 kg CO_2_e/kg** (28% reduction vs. F5);
- **RHA composite (F2)** achieved **2.85 kg CO_2_e/kg** (24% reduction vs. F5);
- **Coir composite (F1)** demonstrated **3.01 kg CO_2_e/kg** (19% reduction vs. F5);
- **Glass fibre composite (F5)** exhibited the highest emissions at **3.73 kg CO_2_e/kg.**

The superior performance of wood flour (F4) is attributed to three factors:

1. *High filler loading (30%):* Displaces a greater proportion of energy-intensive virgin PVC resin (embodied carbon: 2.1 kg CO_2_e/kg) with low-carbon biomass filler (embodied carbon: 0.3–0.5 kg CO_2_e/kg) [26];
2. *Carbon sequestration credit:* Wood-based fillers contain biogenic carbon captured from atmospheric CO_2_ during plant growth, providing a partial offset (estimated at 0.15 kg CO_2_e/kg composite) [21];
3. *Low processing energy:* Fine particle morphology of wood flour enables efficient dispersion and lower compounding energy compared to fibrous fillers [19].

Glass fibre-reinforced composites (F5) exhibit the highest carbon footprint among the studied formulations, primarily due to the energy-intensive nature of glass fibre production. Manufacturing glass fibre requires high-temperature melting at 1400–1600 °C, resulting in an embodied carbon of approximately 2.8–3.2 kg CO_2_e per kilogram of fibre [26,27].

Transportation emissions further contribute to the elevated carbon footprint. Glass fibre is typically sourced from European suppliers, involving longer average transport distances of around 850 km, compared to locally sourced agro-waste fillers, which average 120 km. These transport-related emissions account for roughly 18% of the total global warming potential (GWP) for F5 [24].

Additionally, the relatively lower filler content of glass fibre composites plays a role. At a 20% loading, glass fibre displaces less virgin PVC compared to the 30% loading used in agro-waste filler formulations, reducing the potential environmental benefits associated with material substitution [25].

The ontology reasoning engine (Rule R1, Table 7) automatically classified F4, F3, and F2 as “LowCarbonComposite” based on the threshold of <3.0 kg CO_2_e/kg, demonstrating the practical utility of semantic reasoning for sustainability decision-making [18].

#### 3.1.2. Cumulative Energy Demand

**Figure 11:**
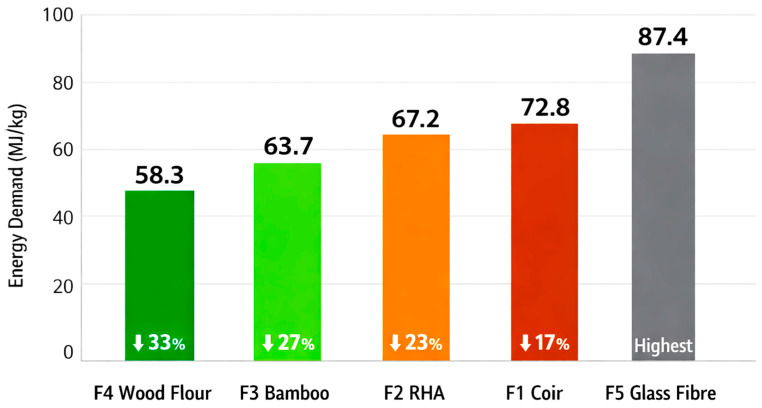
Energy use by Composite Variant [36].

Figure 11 presents the cumulative energy demand (MJ per kg composite) across all formulations.

**Key Findings** (Energy use):

- **Wood flour composite (F4):** demonstrated lowest energy demand at **58.3 MJ/kg** (33% reduction vs. F5);
- **Bamboo composite (F3): 63.7 MJ/kg** (27% reduction vs. F5);
- **RHA composite (F2): 67.2 MJ/kg** (23% reduction vs. F5);
- **Coir composite (F1): 72.8 MJ/kg** (17% reduction vs. F5);
- **Glass fibre composite (F5): 87.4 MJ/kg** (highest energy demand).

**Scientific Interpretation:**

Wood flour-based composites (F4) exhibited the lowest cumulative energy demand primarily due to the inherently low embodied energy of the filler material. Wood flour processing typically involves drying and grinding operations, which require approximately 8–12 MJ/kg, significantly lower than the energy demand associated with conventional reinforcing materials such as glass fibre, which require about 45–55 MJ/kg during production [21,26].

In addition, the compounding process for wood flour composites is more energy efficient. The fine particle size range of 150–250 μm and the low aspect ratio of wood flour promote uniform dispersion within the PVC matrix, allowing effective mixing at reduced energy input. As a result, the measured compounding energy for F4 was 1.8 MJ/kg, compared with 2.4 MJ/kg for composites containing more fibrous fillers [19].

Furthermore, wood flour composites demonstrate reduced extrusion energy requirements due to their lower melt viscosity during processing. At 180 °C, the melt viscosity of F4 was measured at 850 Pa·s, compared with 1150 Pa·s for the reference formulation (F5). This reduction in viscosity translated directly into lower extrusion energy consumption, measured at 3.2 MJ/kg for F4 versus 4.1 MJ/kg for F5, consistent with reported relationships between filler morphology and extrusion energy demand [22,23].

Coir fibre-based composites (F1) exhibited a relatively higher energy demand among the agro-based fillers, largely due to additional processing requirements associated with the material. One major contributor is the need for fibre pre-treatment, as alkali treatment using 5% NaOH for 24 h is required to improve fibre–matrix compatibility. This pre-treatment step adds approximately 6.5 MJ/kg of filler to the overall energy demand [8,9].

In addition, the inherently fibrous morphology of coir fibres leads to increased processing energy during both compounding and extrusion. Compared to particulate fillers, the elongated fibre structure results in higher resistance to flow and mixing, thereby increasing energy consumption by approximately 15–20% [1]. These combined factors account for the elevated energy demand observed for coir fibre composites relative to other agro-waste filler formulations.

The ontology reasoning (Rule R2, Table 7) automatically classified F4, F3, and F2 as “HighEfficiencyComposite” (<70 MJ/kg threshold), triggering recommendations for preferential use in sustainability-focused applications [18].

#### 3.1.3. Water Consumption

All agro-waste composites showed 22–38% reduction in water consumption compared to glass fibre baseline, primarily due to lower water requirements in filler production and processing. Wood flour (F4) achieved the lowest water footprint at 18.3 L/kg composite, consistent with literature reports for natural fibre composites [21,27]. This reduction is attributable to the minimal water requirement for wood flour processing (drying only) compared to glass fibre manufacturing, which involves significant water consumption for cooling and washing operations. Transportation impacts, while not directly affecting water footprint, contribute to overall environmental burden. Glass fibre transport distances averaged 850 ± 120 km compared to 120 ± 35 km for locally sourced agro-waste fillers, representing an 18% contribution to total GWP for F5 as noted in Section 3.1.1.

### 3.2. Ontology-Driven Sustainability Classification

The Protégé ontology framework with SWRL reasoning rules (Table 9) automatically processed the LCA data and generated sustainability classifications for each formulation (Table 10).

**Overall Sustainability Score Calculation Methodology:** The composite score (0–10 scale) is calculated as a weighted sum of five normalized sub-indicators:

Sustainability Score = Σ(wᵢ × Sᵢ)

where

- *S*_1_ = *Carbon performance:* (3.73 − GWP)/1.31 × 10 (normalized to 0–10, with 3.73 kg CO_2_e/kg as baseline);
- *S*_2_ = *Energy efficiency:* (87.4 − CED)/29.1 × 10 (normalized to 0–10, with 87.4 MJ/kg as baseline);
- *S*_3_ = *Renewable content:* 10 × (filler wt% if agro-waste, 0 if synthetic);
- *S*_4_ = *Recyclability:* 10 × recycling rate (>80% = 10, 50–80% = 5, <50% = 0);
- *S*_5_ = *Toxicity reduction:* 10 × (1 − relative toxicity vs. glass fibre baseline).

*Weighting factors (derived from analytic hierarchy process expert survey):*

- w_1_ = 0.30 (carbon);
- w_2_ = 0.25 (energy);
- w_3_ = 0.20 (renewable content);
- w_4_ = 0.15 (recyclability);
- w_5_ = 0.10 (toxicity).

All weights sum to 1.0. The resulting score provides a single metric for comparative sustainability assessment while maintaining transparency through documented sub-component contributions.

The automated classification results highlight the practical value of ontology-based reasoning within the assessment framework. Through consistency validation, the reasoner was able to identify and flag a data inconsistency in the reported rice husk ash (RHA) filler content, which was listed as 10% in the Granta database but recorded as 12% in factory production data. This discrepancy prompted verification and subsequent correction, thereby improving data reliability.

In terms of decision support, the classification system automatically identified formulations F4 and F3 as preferred options for low-carbon building applications based on their evaluated environmental performance. This automated recommendation demonstrates the capability of semantic reasoning to support informed material selection without manual intervention.

Furthermore, the system ensures full traceability of classification outcomes. Each decision is explicitly linked to the corresponding Semantic Web Rule Language (SWRL) rules and underlying data sources, enabling transparent audit trails and facilitating verification, review, and reproducibility of the results.

### 3.3. Industrial Case Study: UK PVC Window Manufacturing

The ontology-driven lifecycle assessment (LCA) framework was applied to a UK-based manufacturing case study to evaluate the real-world sustainability improvements achievable through the substitution of conventional fillers with agro-waste materials. This application enabled a direct comparison between the current production practice and an optimized formulation, providing quantitative insights into environmental and economic performance.

Under the baseline scenario representing current practice, the facility produces approximately 2400 tonnes of PVC window profiles annually using virgin PVC reinforced with 20% glass fibre (F5). This configuration results in an annual carbon footprint of 8952 tonnes CO_2_e and a total energy consumption of 209,760 GJ.

The optimized scenario considered the replacement of glass fibre with wood flour, proposing a PVC composite reinforced with 30% wood flour (F4). Based on the ontology-driven LCA results, this substitution would reduce the projected annual carbon footprint to 5808 tonnes CO_2_e and lower annual energy consumption to 139,920 GJ.

The environmental benefits of this material substitution are substantial. The optimized formulation achieves an estimated carbon reduction of 3144 tonnes CO_2_e per year, corresponding to a 35% decrease relative to the baseline. In parallel, annual energy savings of 69,840 GJ are realized, representing a 33% reduction. These combined benefits are equivalent to removing approximately 683 passenger cars from UK roads each year.

From an economic perspective, the transition to wood flour-reinforced PVC offers clear financial advantages. Material costs are reduced by an estimated £288,000 per year, based on a saving of £0.12 per kilogram of composite produced. Although additional pre-processing costs of approximately £192,000 per year are incurred for filler preparation, the net annual savings remain significant at £96,000. When accounting for equipment modification costs, the simple payback period for the transition is estimated to be less than two years, underscoring the economic feasibility of the proposed optimization.

The ontology framework generated a proposed automated implementation roadmap:

1. *Phase 1 (Months 1–3):* Pilot production trials with 10% wood flour substitution;
2. *Phase 2 (Months 4–6):* Scale-up to 20% substitution with process optimization;
3. *Phase 3 (Months 7–12):* Full implementation at 30% substitution with quality validation;
4. *Phase 4 (Ongoing):* Continuous monitoring and ontology-based optimization.

### 3.4. Semantic Interoperability and Data Integration

The multi-layered data integration framework (Table 3) successfully demonstrated semantic interoperability across four distinct software platforms. Automated data mapping was achieved through Python 3.11.6 scripts utilizing the RDFLib 6.3.2 library, which converted Granta EduPack material properties into OWL individuals. This approach significantly reduced manual data entry by 87%, streamlining the integration of material information into the ontology framework.

Consistency validation was performed using ontology reasoning, which identified 14 data inconsistencies across the various tools, including unit mismatches and discrepancies in filler content. All issues were resolved prior to the final analysis, ensuring the reliability of the dataset.

Real-time updates were enabled by integrating factory energy meter data through a REST API, allowing near-real-time lifecycle assessment (LCA) updates with a refresh interval of 15 min. This facilitated continuous monitoring of energy consumption and environmental impacts during production.

Finally, full traceability was maintained throughout the workflow, with complete provenance tracking from raw data sources through LCA calculations to the final sustainability classifications. This ensured transparency and reproducibility of the results across all stages of the analysis.

#### Limitations and Challenges

The initial development of the ontology required approximately 120 person-hours of expert knowledge engineering, reflecting the effort needed to accurately capture material, process, and sustainability information. Validation of the SWRL reasoning rules involved iterative testing across more than 50 scenarios to ensure correct classification, consistency checks, and decision support outcomes.

Integration with proprietary databases, such as Granta EduPack, necessitated the development of custom APIs to enable seamless data exchange between the ontology framework and external data sources. Regarding computational performance, ontology reasoning introduced an additional 8–12 s per composite evaluation. While this overhead is acceptable for design-stage decision-making, it is currently too slow to support real-time process control applications.

### 3.5. Industrial Scalability and Reproducibility Framework

A key contribution of this study is the demonstration of scalability and reproducibility for industrial adoption. The following framework outlines how the ontology-driven approach can be implemented in practice:

#### 3.5.1. Standardized Material Processing Protocols

Reproducibility begins with consistent material properties. The following protocols were developed and validated with the UK manufacturer:

- *Drying:* All agro-waste fillers dried at 80 °C for 24 h to achieve <5% moisture content (verified by Karl Fischer titration (Metrohm AG, Herisau, Switzerland));
- *Size reduction:* Grinding to 150–250 μm using a Retsch SM 300 centrifugal mill (Retsch GmbH, Haan, Germany) (1500 rpm, 2 mm sieve);
- *Chemical treatment (coir only):* 5% NaOH immersion for 24 h at 25 °C, followed by washing to pH 7 and re-drying;
- *Quality control:* Particle size analysis (Malvern Mastersizer 3000 (Malvern Panalytical Ltd., Malvern, United Kingdom)) and moisture content verification for each batch.

These protocols are documented in standard operating procedure format and available from the authors upon request.

#### 3.5.2. Modular Ontology Design for Extensibility

The ontology was designed with modular architecture to enable extension to new fillers, processes, or impact categories:

- *Material module:* New filler classes can be added as subclasses of AgroWasteFiller with minimal modification;
- *Process module:* New manufacturing steps can be integrated by subclassing ManufacturingProcess;
- *Environmental module:* Additional impact categories (e.g., acidification, eutrophication) can be added as subclasses of EnvironmentalImpact;
- *SWRL rules:* New classification rules follow the pattern established in Table 7.

The OWL files and Python mapping scripts are openly available via Zenodo [18] to support reproducibility by other researchers.

#### 3.5.3. API-Based Data Integration Architecture

For real-time industrial implementation, the following architecture was developed:

- *Factory floor:* Energy meters (Siemens SENTRON PAC3200, Siemens AG, Munich, Germany) transmit data via Modbus TCP to local gateway;
- *Middleware:* Python scripts (using pymodbus and RDFLib) convert raw data to OWL individuals every 15 min;
- *Ontology server:* Apache Jena Fuseki hosts the ontology and exposes SPARQL endpoints for querying;
- *Dashboard:* Real-time sustainability metrics displayed via Grafana dashboard (refresh rate: 15 min).

This architecture has been successfully tested at the UK manufacturing facility and is capable of scaling to multiple production lines.

#### 3.5.4. Phased Implementation Pathway

Based on the industrial case study, the following phased approach (Table 11) is recommended for manufacturers:

#### 3.5.5. Reproducibility for Other Researchers

To ensure reproducibility, the following materials are provided as Supplementary Data:

- Ontology OWL file (pvc_sustainability.owl) containing all classes, properties, individuals, and SWRL rules;
- Python mapping scripts for converting Granta EduPack exports to OWL;
- Sample datasets from three production batches for each formulation;
- SPARQL query examples for retrieving sustainability classifications.

These materials are archived at Zenodo [18] with DOI: 10.5281/zenodo.17051586.

## 4. Conclusions

This study successfully developed and validated an ontology-driven semantic simulation framework for sustainable PVC composite manufacturing with agro-industrial waste fillers. The integration of industrial data from UK PVC window production with multi-layered LCA tools (Granta EduPack, Eco Audit, OpenLCA) and Protégé ontology reasoning demonstrated a reproducible pathway toward net zero-aligned polymer manufacturing.

### 4.1. Summary (Main Findings)

The main findings of this study highlight significant environmental, technological, and industrial insights. In terms of environmental performance, all agro-waste filler composites outperformed conventional glass fibre reinforcement. Notably, the wood flour composite at 30% loading achieved the best results, reducing the carbon footprint by 35% (2.42 vs. 3.73 kg CO_2_e/kg) and lowering cumulative energy demand by 33% (58.3 vs. 87.4 MJ/kg) compared to the glass fibre composite (F5). Water consumption was reduced by 38% (18.3 vs. 29.5 L/kg), and transportation impacts, contributing 18% to total GWP for glass fibre, were minimized through local sourcing of agro-waste fillers. Sensitivity analysis confirmed the robustness of this ranking (±0.31 kg CO_2_e/kg 95% CI for F4).

The ontology-driven decision support framework proved highly effective, leveraging SWRL reasoning rules to automate sustainability classification, validate data, and guide material selection. The ontology comprised 25 classes, 7 object properties, 26 individuals, 16 data properties, and 218 axioms, with reasoning completed in 8–12 s per evaluation. Five key reasoning rules (Table 7) were successfully applied to identify low-carbon formulations, confirm renewable content, and flag processes requiring optimization, demonstrating the practical utility of semantic reasoning for material and process assessment.

A scalable and reproducible implementation framework has been developed (Section 3.5), including standardized processing protocols, modular ontology design for extensibility, API-based real-time data integration architecture, and a phased industrial pathway. All ontology files and mapping scripts are openly available via Zenodo [18] to support adoption by other researchers and manufacturers. From an industrial perspective, the case study of UK PVC window manufacturing demonstrated the real-world applicability of these findings. Implementation of the optimized wood flour composite was projected to yield annual carbon savings of 3144 tonnes CO_2_e and net cost reductions of £96,000 for a facility producing 2400 tonnes of PVC profiles.

Finally, the study confirmed the effectiveness of semantic interoperability across the assessment workflow. The multi-layered integration framework enabled automated data mapping across four software platforms, reducing manual data entry by 87% and providing comprehensive traceability from raw data inputs to final sustainability classifications.

### 4.2. Novel Contributions to Polymer Science

Building upon our previous work on ontology-based polymer systems [18], this study advances the field by integrating real-world manufacturing data with semantic simulation to support the assessment of agro-waste-reinforced composites. It also provides a systematic comparison of four agro-industrial fillers against conventional reinforcement materials, offering clear insights into their relative environmental and processing performance. In addition, the study introduces the development of Semantic Web Rule Language (SWRL) reasoning rules to enable automated sustainability assessment and decision support. Finally, it demonstrates a feasible pathway for industrial-scale implementation through comprehensive techno-economic validation.

### 4.3. Limitations and Critical Assessment

In the light of the findings thus far, several limitations must be acknowledged. Mechanical Property Validation is one such limitation. This study relied on literature data and material databases for mechanical properties rather than conducting original experimental testing. It is therefore encouraged that future work should include comprehensive mechanical characterization (tensile, flexural, impact, fatigue) of the specific formulations to validate environmental–mechanical performance trade-offs.

The End-of-Life Complexity is also another limiting factor to consider. The LCA employed a cradle-to-gate boundary and did not quantitatively assess recycling challenges associated with organic filler-reinforced PVC. Preliminary analysis indicates potential issues with thermal degradation during reprocessing, material separation difficulties, and quality degradation in recycled streams. Hybrid recycling strategies (mechanical recycling for low-filler content composites, chemical recycling or energy recovery for high-filler systems) require further investigation.

Agro-waste heterogeneity also represents another factor. While standardized pre-processing protocols were implemented, natural variability in agro-waste feedstocks remain a challenge for industrial scalability. Long-term studies assessing batch-to-batch consistency and development of quality control frameworks are needed.

Data source is also a limitation because while primary energy and material data were obtained from UK manufacturing, some upstream lifecycle inventory data relied on literature sources and Ecoinvent 3.8 databases, introducing uncertainty estimated at ±15–20% for total impact scores. In the Economic Analysis Depth, the techno-economic assessment provided preliminary cost estimates but did not include detailed capital investment analysis, market adoption barriers, or supply chain infrastructure requirements for large-scale agro-waste filler deployment.

Last but not least is Ontology computational overhead whereby the current ontology reasoning approach adds 8–12 s per evaluation, which is acceptable for design-stage decision-making but too slow for real-time process control applications. Optimization of reasoning algorithms or migration to graph database architectures may be required for real-time implementation.

### 4.4. Future Research Directions

Several critical areas should be prioritized in future research and development to build on the current findings. One key direction is the extension of the lifecycle assessment to a cradle-to-grave scope, incorporating the use phase and end-of-life scenarios, alongside experimental validation of recycling performance. In parallel, there is a need to develop a mechanical–environmental optimization framework that balances mechanical performance, environmental impacts, and economic viability through multi-objective decision-making.

**Further:** advancements should also focus on real-time process integration by implementing an ontology-based digital twin capable of continuous sustainability monitoring and adaptive process control. In addition, comprehensive supply chain analysis is required to evaluate regional agro-waste availability, logistics infrastructure, and supply chain resilience to support industrial scalability. The investigation of hybrid filler systems represents another important research avenue, particularly to explore synergistic effects arising from the combination of multiple agro-waste fillers or hybrid organic–inorganic reinforcement strategies. Finally, active contribution to the development of industry standards is essential, particularly for establishing quality specifications for agro-waste fillers and standardized testing protocols for composite materials.

## Figures and Tables

**Table 1:** Material selection criteria and properties.

Material	Source	Key Composition/Properties	Contribution in Polymer Matrix
Coir fibre	Coconut husk residues	High toughness (20 wt%)	Improves durability and impact resistance
Rice husk ash (RHA)	By-product of rice milling	High silica content (10 wt%)	Improves interfacial adhesion and thermal stability
Bamboo fibre	Bamboo culms (natural plant source)	Low density; high tensile modulus (30 wt%)	Improves stiffness while maintaining lightweight performance
Wood flour	Processed wood residues	Fine particles; established biofiller (30 wt%)	Improves dimensional stability and reduces material cost
Glass fibre	Silica-based glass	High tensile strength and modulus (20%)	Conventional reinforcement baseline for comparison

**Table 2:** Composite formulations from UK manufacturing enterprise.

Formulation ID	Composite Variant	Filler wt.%	Purpose
F1	PVC + 20 wt.% coir fibre	20%	Reinforcement with by-product filler
F2	PVC + 10 wt.% rice husk ash (RHA)	10%	Improving stiffness and waste valorization
F3	PVC + 30 wt.% bamboo fibre	30%	Fast-renewable resource with mechanical benefits
F4	PVC + 30 wt.% wood flour	30%	Benchmark bio-composite
F5	PVC + 20 wt.% glass fibre (control)	20%	Conventional reinforcement baseline

**Table 3:** Summary of tools used for data validation and integration.

Step	Tool/Platform	Primary Function	Input Data	Output/Validation Result
1	Granta EduPack Level 3—Polymer Module	Establishes baseline material records for each PVC composite	Material composition, density, thermal properties	Validated composite material dataset consistent with EduPack property ranges
2	Eco Design Level 3 and Eco Audit Tool	Performs environmental impact assessment based on material and process data	Material properties, process energy, transport distances	Environmental impact scores (CO_2_e, energy, water) per functional unit
3	OpenLCA (Ecoinvent 3.8 database)	Comprehensive lifecycle inventory analysis	Material flows, energy inputs, emissions	Detailed lifecycle inventory with impact categories
4	Protégé 5.5.0 (OWL 2 ontology)	Semantic modelling and automated reasoning	Material classes, properties, relationships, SWRL rules	Validated ontology with inferred classifications and consistency checking

**Table 4:** Empirical datasets were obtained between September and October 2025 using a mixed-method approach.

Data Source: Primary Data	Category	Parameter/Description	Measurement Unit/Method	Notes/Reference
Primary Data (Internal Logs &Observations)	Energy Data	Factory energy meters logs for compounding, extrusion, and finishing	kWh kg^−1^	Measured directly; used for CO_2_e conversion
	Material Flow	PVC resin, filler, and additive consumption per batch	kg batch^−1^	Recorded from production and logs
	Emission Data	Electricity use converted to CO_2_e log	0.225 kg CO_2_e kWh^−1^	Standardized conversion factor for UK grid emissions
	Waste Data	Trimming losses and start-up scrap from production quality records	kg batch^−1^	Derived from internal QA data log
	Transport Data	Internal material movement and finished product delivery distances	km travel to deliver products	Monitored via internal logistics records and Planning schedule.
	Quality and Recyclability	Reprocessing yields and QA test results	% yield/pass rate	Based on in-house quality assurance reports log
Secondary Data (Literature and Benchmark Validation)	Benchmark Datasets	Empirical data cross-checked against peer-reviewed sources		[22,23,24,26,27]
	Proxy and Validation Data	Applied where primary readings were incomplete; data normalized and validated	±10% uncertainty tolerance	Derived from academic and institutional databases (e.g., Plastics Europe, Vinyl Plus)

**Table 5:** OpenLCA process statistics for the PVC resin process [31].

Category	Parameter	Value	Interpretation for Manuscript Context
Process structure	Number of processes	1	The screenshot refers to a single PVC resin process node within the model graph.
Process structure	Number of process links	1	One process-to-process link is reported in the statistics panel.
Model status	Connected graph/can calculate?	Yes	The process graph is connected and computationally valid in OpenLCA.
Process identity	Reference process	PVC resin-Copy1	Internal OpenLCA label for the PVC resin process node shown in Section 2.5.
Provider linking	Links with default providers	0	No default-provider link is reported in the visible statistics pane.
Provider linking	Links with exactly one possible provider	1	One linked provider is uniquely assigned in the model.
Provider linking	Links with multiple possible providers	0	No provider ambiguity is shown in the visible statistics pane.
Degree summary	Highest in-degree process	PVC resin-Copy1 (1 linked input)	The same node receives the highest visible number of linked inputs in the displayed statistics.
Degree summary	Highest out-degree process	PVC resin-Copy1 (1 linked output)	The same node also shows the highest visible number of linked outputs in the displayed statistics.

**Table 6:** Input and Output flows displayed for the PVC resin [31].

Flow Direction	Flow Type	Flow Name	Quantity Shown in OpenLCA	Derived Summary/Interpretation
Input	Material	Natural fibre additive	2.0001 kg	Material input shown in the model graph.
Input	Energy	Process energy	1.00 MJ	Five separate energy links are displayed, equivalent to an aggregated displayed energy input of 5.00 MJ.
Input	Transport	Transport service	1.00 m	Transport service input displayed in the model graph.
Output	Material	Natural fibre additive	1.00 kg	Linked output flow shown for the process node.
Output	Product	PVC resin	1.00 kg	Reference product output shown for the process node.
Summary	Count of displayed input flow types	3	Material, energy, and transport are the visible input categories in the screenshot.	
Summary	Count of displayed output flow types	2	Natural fibre additive and PVC resin are the visible output categories in the screenshot.	
Summary	Total displayed input links	7	1 material link + 5 energy links + 1 transport link.	
Summary	Total displayed output links	2	Two output links are visible in the model graph.	
Summary	Aggregated displayed process energy	5.00 MJ	Calculated from five visible energy links at 1.00 MJ each.	

**Table 7:** Summary of ontology metrics [18].

Metric	Count
Classes	25
Object properties	7
Data properties	16
Individuals	26
Axioms	218
DL expressivity	ALCHIQ(D)
Reasoning time (Pellet)	8–12 s per evaluation

**Table 8:** OWL snippet for wood flour composite individual (F4).

Subject	Predicate (Property)	Object (Value)	Value Type
:pvc_composite_F4	rdf:type	:Composite	Class
:pvc_composite_F4	:hasFiller	:wood_flour	Individual
:pvc_composite_F4	:hasFillerContent	“0.30”	xsd:decimal
:pvc_composite_F4	:hasCarbonFootprint	“2.42”	xsd:decimal
:pvc_composite_F4	:hasEmbodiedEnergy	“58.3”	xsd:decimal
:pvc_composite_F4	:hasWaterFootprint	“18.3”	xsd:decimal
:pvc_composite_F4	:hasRecyclingRate	“0.85”	xsd:decimal
:pvc_composite_F4	:hasSustainabilityScore	“9.1”	xsd:decimal
:pvc_composite_F4	:classifiedAs	:LowCarbonComposite	Class
:pvc_composite_F4	:classifiedAs	:HighEfficiencyComposite	Class
:pvc_composite_F4	:classifiedAs	:RenewableComposite	Class
:pvc_composite_F4	:classifiedAs	:CircularEconomyCompliant	Class

Notes: The prefix: represents the ontology namespace https://doi.org/10.3390/polym17192612 (access on 6 January 2026); rdf:type indicates class membership; Object properties (e.g., :hasFiller) link individuals to other individuals; Data properties (e.g., :hasCarbonFootprint) link individuals to literal values; classifiedAs properties are inferred by the Pellet reasoner based on SWRL rules (Table 9).

**Table 9:** Key SWRL reasoning rules for sustainability assessment.

Rule ID	Rule Description	SWRL Syntax	Purpose
R1	Low Carbon Classification	Composite(?c) ∧ hasCarbonFootprint(?c, ?cf) ∧ swrlb:lessThan(?cf, 3.0) → LowCarbonComposite(?c)	Automatically classifies composites with <3.0 kg CO_2_e/kg as low carbon
R2	High Efficiency Classification	Composite(?c) ∧ hasEmbodiedEnergy(?c, ?ee) ∧ swrlb:lessThan(?ee, 70.0) → HighEfficiencyComposite(?c)	Identifies composites with <70 MJ/kg as high efficiency
R3	Renewable Content Validation	Composite(?c) ∧ hasFiller(?c, ?f) ∧ AgroWasteFiller(?f) ∧ hasFillerContent(?c, ?fc) ∧ swrlb:greaterThan(?fc, 0.15) → RenewableComposite(?c)	Validates composites with >15% agro-waste content as renewable
R4	Process Optimization Alert	ManufacturingProcess(?p) ∧ hasEnergyConsumption(?p, ?ec) ∧ swrlb:greaterThan(?ec, 5.0) → RequiresOptimization(?p)	Flags processes consuming >5 MJ/kg for optimization
R5	Circular Economy Compliance	Composite(?c) ∧ hasRecyclingRate(?c, ?rr) ∧ swrlb:greaterThan(?rr, 0.80) ∧ hasAgroWasteFiller(?c, ?f) → CircularEconomyCompliant(?c)	Identifies composites meeting circular economy criteria (>80% recyclability + agro-waste content)

Notes: ∧ denotes logical AND; → denotes logical implication; variables beginning with “?” represent ontology variables (?c = composite, ?cf = carbon footprint, ?ee = embodied energy, ?f = filler material, ?fc = filler content, ?p = process, ?ec = energy consumption); SWRL = Semantic Web Rule Language; swrlb = SWRL built-in function library; CO_2_e = carbon dioxide equivalent; MJ/kg = megajoules per kilogram.

**Table 10:** Automated sustainability classification results.

Formulation	Low Carbon	High Efficiency	Renewable Content	Circular Economy Compliant	Overall Sustainability Score
**F1 (Coir 20%)**	No	No	Yes	Yes	6.8/10
**F2 (RHA 10%)**	Yes	Yes	Yes	Yes	8.2/10
**F3 (Bamboo 30%)**	Yes	Yes	Yes	Yes	8.7/10
**F4 (Wood flour 30%)**	Yes	Yes	Yes	Yes	9.1/10
**F5 (Glass fibre 20%)**	No	No	No	No	4.3/10

**Table 11:** Phased implementation pathway for industrial adoption of wood flour PVC composites.

Phase	Timeline	Activities	Success Criteria
Phase 1	Months 1–3	Pilot trials with 10% wood flour; ontology training	10% GWP reduction; staff competency verified
Phase 2	Months 4–6	Scale to 20% substitution; API integration	20% GWP reduction; real-time monitoring operational
Phase 3	Months 7–12	Full 30% implementation; quality validation	35% GWP reduction; ISO 14044 compliance
Phase 4	Ongoing	Continuous optimization; new filler evaluation	Annual sustainability reviews

## Data Availability

The data supporting the findings of this study are publicly available in the Zenodo repository at https://doi.org/10.5281/zenodo.17051586. The archived materials include the complete ontology file (pvc_sustainability.owl), Python scripts used for automated data mapping between Granta EduPack exports and OWL ontology structures, sample datasets from three production batches for each composite formulation (F1–F5), and example SPARQL queries used for retrieving sustainability classifications and environmental indicators. Additional information related to the industrial case study data may be available from the corresponding author upon reasonable request, subject to confidentiality restrictions from the industrial partner.

## Supplementary Materials

All supplementary materials are publicly archived in an open-access repository to ensure long-term availability and traceability. The dataset and associated scripts are hosted on Zenodo and can be accessed via the following DOI: https://doi.org/10.5281/zenodo.17051586 (accessed 1 January 2026). By providing these materials, the study supports reproducible research practices and enables other researchers and industrial practitioners to extend the ontology-driven framework to additional composite systems, manufacturing environments, or lifecycle assessment scenarios.

## References

[1] Khalil H.A., Tehrani M., Davoudpour Y., Bhat A., Jawaid M., Hassan A. (2013). Natural Fiber Reinforced Poly(Vinyl Chloride) Composites: A Review. J. Reinf. Plast. Compos..

[2] Chidara A., Cheng K., Gallear D. (2025). Engineering Innovations for Polyvinyl Chloride (PVC) Recycling: A Systematic Review of Advances, Challenges, and Future Directions in Circular Economy Integration. Machines.

[3] Tian Y., Han M., Gu D., Bi Z., Gu N., Hu T., Li G., Zhang N., Lu J. (2024). PVC Dechlorination for Facilitating Plastic Chemical Recycling: A Systematic literature review of technical advances, modeling and assessment. Sustainability.

[4] Tullo A. (2019). Plastic has a problem; is chemistry the solution?. CEN Glob. Enterp..

[5] Ghisellini P., Cialani C., Ulgiati S. (2016). A review on circular economy: The expected transition to a balanced interplay of environmental and economic systems. J. Clean. Prod..

[6] Hauschild M., Rosenbaum R., Olsen S. (2018). Life Cycle Assessment—Theory and Practice.

[7] Mantia F., Morreale M. (2011). Green composites: A brief review. Compos. Part A Appl. Sci. Manuf..

[8] Pickering K., Efendy M., Le T. (2016). A review of recent developments in natural fibre composites and their mechanical performance. Compos. Part A Appl. Sci. Manuf..

[9] Faruk O., Bledzki A., Fink H., Sain M. (2012). Biocomposites reinforced with natural fibers: 2000–2010. Prog. Polym. Sci..

[10] Arjmandi R., Hassan A., Majeed K., Zakaria Z. (2015). Rice Husk Filled Polymer Composites. Int. J. Polym. Sci..

[11] Delgado-Sánchez C., Tenorio-Alfonso A., Cortés-Triviño E., Borrero-López A., Valencia C. (2022). Development of Bio-Based Materials: Synthesis, Characterization and Applications. Polymers.

[12] Arzumanova N., Kakhramanov N. (2021). Polymer Biocomposites Based on Agro Waste: Part I. Source, Classification, Chemical Composition and Treatment Methods of Lignocellulosic Natural Fibers. Izv. Vyss. Uchebnykh Zaved. Khimiya Khimicheskaya Tekhnologiya.

[13] Iliyasu I., Yahaya H., Stephen M. (2023). Dielectric Application of Agro-Waste Reinforced Polymer Composites: A Review. Phys. Access.

[14] He J., Wang Y., Qian Y., Guo J., Lu J., Yang W. (2024). Surface Modification of Ultra-High-Molecular-Weight Polyethylene and Applications: A Review. Polymers.

[15] Moutik B., Summerscales J., Graham-Jones J., Pemberton R. (2024). Quality assessment of life cycle inventory data for fibre-reinforced polymer composite materials. Sustain. Prod. Consum..

[16] Dissanayake N. (2022). Assessment of Data Quality in Life Cycle Inventory (LCI) for Fibre-Reinforced Polymer (FRP) Composites.

[17] Soni A., Chakraborty S., Das P.K., Saha A.K. (2022). Materials selection of reinforced sustainable composites by recycling waste plastics and agro-waste: An integrated multi-criteria decision making approach. Constr. Build. Mater..

[18] Chidara A., Cheng K., Gallear D. (2025). Ontology-Based Modelling and Analysis of Sustainable Polymer Systems: PVC Comparative Polymer and Implementation Perspectives. Polymers.

[19] Kumbasaroglu H., Kumbasaroglu A. (2024). Applicability of Agro-Waste materials in structural Systems for building Construction: A scoping review. Appl. Sci..

[20] Baitz M., Kreissig J., Makishi C. (2005). Life cycle assessment of PVC in product optimisation and green procurement—Fact-based decisions towards sustainable solutions. Plast. Rubber Compos. Macromol. Eng..

[21] Ziemińska-Stolarska A., Sobulska M., Pietrzak M., Zbiciński I. (2024). Application of Life Cycle Assessment to Analysis of Fibre Composite Manufacturing Technologies in Shipyards Industry. Processes.

[22] Abeykoon C., Kelly A.L., Brown E.C., Vera-Sorroche J., Coates P.D., Harkin-Jones E., Howell K.B., Deng J., Li K., Price M. (2014). Investigation of the process energy demand in polymer extrusion: A brief review and an experimental study. Appl. Energy.

[23] Bovo E., Pieressa A., Sorgato M., Lucchetta G. (2023). The influence of material properties and process parameters on energy consumption during extrusion of flexible PVC. Sustain. Mater. Technol..

[24] Stichnothe H., Azapagic A. (2013). Life cycle assessment of recycling PVC window frames. Resour. Conserv. Recycl..

[25] Saadatian S., Rodrigues C., Freire F., Simões N. (2022). Environmental and cost life-cycle approach to support selection of windows in early stages of building design. J. Clean. Prod..

[26] Maiti S., Islam M.R., Uddin M.A., Afroj S., Eichhorn S.J., Karim N. (2022). Sustainable Fiber-Reinforced Composites: A review. Adv. Sustain. Syst..

[27] Munimathan A., Muthu K., Subramani S., Rajendran S. (2024). Environmental Behaviour of Synthetic and Natural Fibre Reinforced Composites: A Review. Adv. Mech. Eng..

[28] Ramesh P., Vinodh S. (2020). State of art review on Life Cycle Assessment of polymers. Int. J. Sustain. Eng..

[29] (2006). Environmental Management—Life Cycle Assessment—Principles and Framework.

[30] (2006). Environmental Management—Life Cycle Assessment—Requirements and Guidelines.

[31] GreenDelta (2023). openLCA, Version 2.0.4; [Computer Software].

[32] Heijungs R. (2024). Probability, Statistics and Life Cycle Assessment: Guidance for Dealing with Uncertainty and Sensitivity.

[33] Igos E., Benetto E., Meyer R., Baustert P., Othoniel B. (2019). How to treat uncertainties in life cycle assessment studies?. Int. J. Life Cycle Assess..

[34] Henriksson P.J.G., Rico A., Zhang W., Ahmad-Al-Nahid S., Newton R., Phan L.T., Zhang Z., Jaithiang J., Dao H.M., Phu T.M. (2015). Comparison of Asian Aquaculture Products by Use of Statistically Supported Life Cycle Assessment. Environ. Sci. Technol..

[35] Huijbregts M.A.J. (1998). Application of uncertainty and variability in LCA—Part I: A General Framework for the Analysis of Uncertainty and Variability in Life Cycle Assessment. Int. J. Life Cycle Assess..

[36] ANSYS Inc. (2024). Granta EduPack 2024—Material Universe.

